# The Parasympathetic Root of the Submandibular Ganglion: A Review

**DOI:** 10.7759/cureus.33775

**Published:** 2023-01-14

**Authors:** Edward C. Muo, Juan J Cardona, Arada Chaiyamoon, Joe Iwanaga, R. Shane Tubbs

**Affiliations:** 1 Department of Neurosurgery, Tulane University School of Medicine, New Orleans, USA; 2 Department of Anatomy, Faculty of Medicine, Khon Kaen University, Khon Kaen, THA; 3 Department of Neurology, Tulane University School of Medicine, New Orleans, USA; 4 Department of Oral and Maxillofacial Anatomy, Graduate School of Medical and Dental Sciences, Tokyo Medical and Dental University, Tokyo, JPN; 5 Department of Anatomical Sciences, St. George’s University, St. George’s, GRD; 6 Department of Structural and Cellular Biology, Tulane University School of Medicine, New Orleans, USA; 7 Department of Surgery, Tulane University School of Medicine, New Orleans, USA; 8 Department of Neurosurgery, Ochsner Neuroscience Institute, Ochsner Health System, New Orleans, USA

**Keywords:** clinical, anatomy, secretion, saliva, salivary gland, submandibular ganglion

## Abstract

The submandibular ganglion is a small fusiform-shaped cluster of cell bodies of the parasympathetic nervous system. Parasympathetic innervation of the submandibular gland is not only responsible for the secretion of saliva, but it also plays a main role in the development and regeneration of the gland. The parasympathetic root of the submandibular ganglion or the posterior branch of the lingual nerve to the submandibular ganglion is one of three roots of the submandibular ganglion. Using standard search engines (PubMed, Google), papers in English discussing the anatomy, embryology, variations, and clinical significance of the parasympathetic root of the submandibular ganglion were reviewed.

## Introduction and background

Anatomy

**Figure 1:**
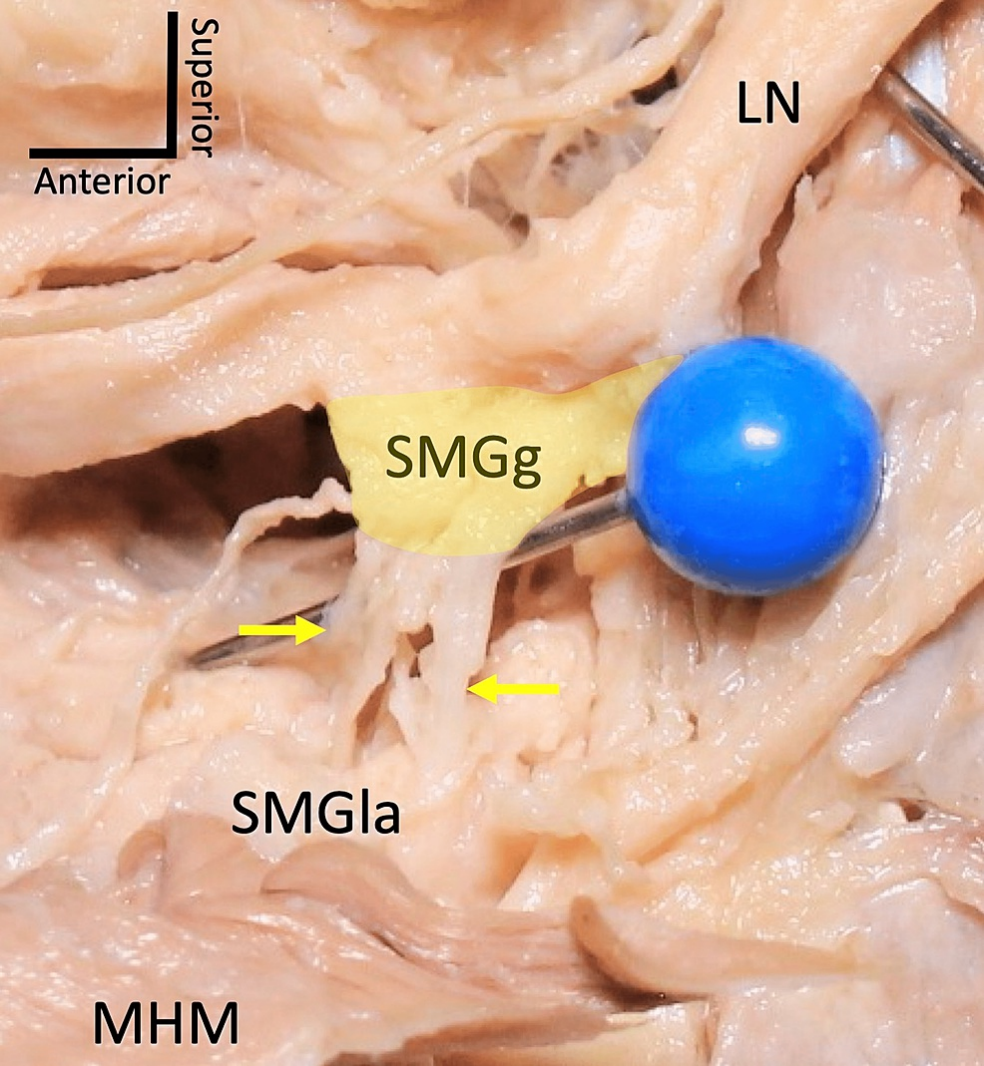
Two branches (arrows) connecting the left submandibular ganglion (SMGg) and the submandibular gland (SMGla). Note that the parasympathetic root of the SMGg from the lingual nerve (LN) is not seen as the LN and SMGg fuse. MHM: mylohyoid muscle. Author's own work.

The submandibular ganglion is a small fusiform-shaped cluster of cell bodies of the parasympathetic nervous system (Figure 1) [1]. The ciliary, pterygopalatine, otic, and submandibular ganglia comprise the four parasympathetic ganglia in the head and neck regions. The submandibular ganglion lies lateral to the hyoglossus muscle, superior to the deep part of the submandibular gland, and is inferior to the lingual nerve.

**Figure 2:**
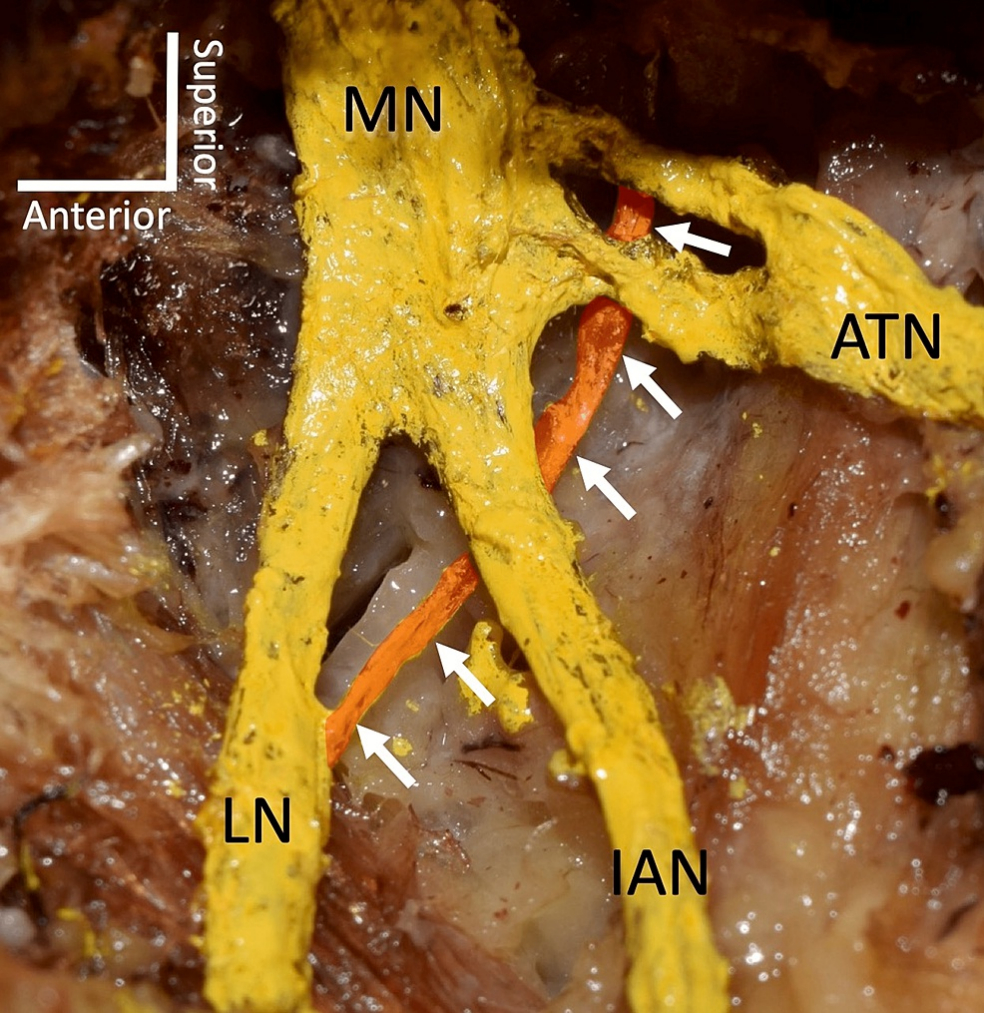
Chorda tympani (arrows) joining the lingual nerve (LN) posteriorly. MN: mandibular nerve; IAN: inferior alveolar nerve; ATN: auriculotemporal nerve. Author's own work.

The parasympathetic root of the submandibular ganglion or the posterior branch of the lingual nerve to the submandibular ganglion is one of three roots of the submandibular ganglion. Parasympathetic fibers originate from the superior salivatory nucleus (SSN) in the pons and are conveyed by the nervus intermedius carrying both sensory and parasympathetic preganglionic fibers of the facial nerve (CN VII). The facial nerve carries these fibers via the facial canal in the middle ear and just before exiting the skull (approximately 5 mm above the stylomastoid foramen), gives off the chorda tympani [2,3]. Preganglionic parasympathetic nerve fibers from the chorda tympani (CN VII) join the lingual nerve posteriorly in the infratemporal fossa (Figure 2) to synapse with postganglionic parasympathetic fibers in the submandibular ganglion and leave to innervate the submandibular and sublingual glands [3,4].

The sympathetic and sensory roots run through the submandibular ganglion, while parasympathetic root fibers are the only fibers to synapse within the submandibular ganglion. The parasympathetic fibers, after synapsing in the submandibular ganglion, extend on to the submandibular and sublingual glands directly (Figure 1). It is assumed that some of these fibers also extend to the minor salivary glands e.g., labial glands. However, the exact pathway for these fibers is not known. Both the parasympathetic and sympathetic parts of the autonomic nervous system innervate all three major salivary glands (parotid, submandibular, and sublingual) to stimulate the secretion of saliva [5]. Parasympathetic innervation of the submandibular gland is not only responsible for the secretion of saliva, but it also plays the main role in development and regeneration of the gland [5,6].

## Review

Neural regulation of the submandibular and sublingual salivary glands

The composition and volume of saliva secreted are controlled by the autonomic nervous system. The parasympathetic root of the submandibular ganglion conveys parasympathetic stimulation to induce the secretion of saliva and contraction of myoepithelial cells of the submandibular and sublingual salivary glands [4]. Cholinergic parasympathetic preganglionic and postganglionic nerves release acetylcholine which results in the activation of M3 and M1 muscarinic receptors in acinar cells of the submandibular and sublingual glands [5,7]. Co-transmitters are also responsible for the parasympathetic response, including vasoactive intestinal peptide (VIP) and nitric oxide which contribute to vasodilation and protein secretion in acinar cells [8-10].

Parasympathetic stimulation results in an increase in the volume of saliva and watery (serous) ion-rich, protein-poor saliva secreted from the salivary glands [4]. Conversely, the sympathetic root of the submandibular ganglion, which has cell bodies in the superior cervical ganglion and postganglionic fibers that run through the submandibular ganglion to reach blood vessels of the submandibular and sublingual glands, releases noradrenaline on B1 adrenoceptors, resulting in vasoconstriction of arteries in the salivary glands and secretion of protein-rich saliva [4,6]. Both parasympathetic and sympathetic stimulation result in the increased salivary secretion of secretory-IgA, an immunoglobin involved in the mucosal anti-microbial response [7].

Parasympathetic innervation is more abundant than sympathetic innervation in the salivary glands and exerts more control over the secretion of saliva throughout the day [10]. The salivary reflex involving the salivatory nuclei in the brainstem is mediated predominantly by parasympathetic effectors. The superior salivatory nuclei, containing efferent preganglionic fibers of the parasympathetic root of the submandibular ganglion, produce changes in secretion and vasodilation of the submandibular and sublingual glands, while the inferior salivatory nuclei produce changes in secretion within the parotid gland.

Stimulation of the parasympathetic root of the submandibular ganglion results in the secretion of saliva from the submandibular glands that produce 60% of saliva volume at rest. The sublingual glands contribute less at 7-8% of resting saliva volume [10]. Combined, the parasympathetic root of the submandibular ganglion is responsible for most of the saliva secretion. Table 1 lists studies that have described the neural pathways for such innervation.

**Table 1:** References about variations and normal innervation of the submandibular and sublingual salivary glands.

Salivary Glands	Variations
Anatomy, Head and Neck, Sublingual and Submandibular Glands, Grewal et al. (2022) [11]	A large sublingual glandular branch of the lingual nerve: a rare case report, Albuck et al. (2022) [12]
Excision of sublingual gland, Ogle (2021) [13]	Dual innervation of the submandibular gland by nerve to mylohyoid and chorda tympani, Ryumon et al. (2021) [14]
Identification of CNS neurons with polysynaptic connections to both the sympathetic and parasympathetic innervation of the submandibular gland, Hettigoda et al. (2015) [15]	Lingual nerve entrapment in fused submandibular and sublingual salivary glands: a unique finding, Nayak and Kumar (2018) [16]
Anatomy, biogenesis and regeneration of salivary glands, Holmberg et al. (2014) [4]	Nerve cell bodies and small ganglia in the connective tissue stroma of human submandibular glands, Tosios et al. (2010) [17]
Innervation and secretory function of transplanted human submandibular salivary glands, Geerling et al. (2008) [18]	Anatomic variation of cranial parasympathetic ganglia, Siéssere et al. (2008) [1]
Transcutaneous excision of the submandibular and sublingual glands: notes on anatomy and surgical technique, Fornaro et al. (2007) [19]	
The surgical anatomy of the sublingual gland, Obradović et al. (1990) [20]	
Innervation of the submandibular gland, Lolli et al. (1989) [21]	
Ultrastructural aspects of the submandibular gland, Kuntz and Richins (1946) [22]

Embryology

Autonomic ganglia, including the submandibular ganglion, are derived from neural crest cells. Neural crest cells migrate and differentiate into Schwann cell precursors prior to becoming cranial parasympathetic ganglia [23,24]. Parasympathetic innervation plays a major role in submandibular gland organogenesis. The submandibular and sublingual glands develop starting at week six of gestation [2,4]. Parasympathetic-epithelial communication is initiated where neural crest-derived parasympathetic precursors wrap around epithelial stalks and coalesce to form the parasympathetic ganglia [2,4,6,7,10]. Growth factors, including neurotrophic factor neurturin (NRTN) and glial cell line-derived neurotrophic factor (GDNF), are important for parasympathetic neuronal survival and increase parasympathetic ganglion function [2,5]. Parasympathetic root innervation also stimulates ductal tubulogenesis of the epithelium of the submandibular gland and is mediated by the release of VIP from parasympathetic nerves, resulting in lumen formation and expansion [25].

Variations

Few variations of the parasympathetic root of the submandibular ganglion have been reported. Siessere et al. reported on variations in the morphology of the four cranial parasympathetic ganglia in forty adult cadavers and noted the variation in the number and volume of parasympathetic nerve fiber bundles attached to the submandibular ganglion and their proximity to the lingual nerve ranging from 2mm to 6mm [1]. While performing a routine dissection on an adult male cadaveric head, Ryumon et al. reported a unique dual innervation of the right submandibular gland via the nerve to mylohyoid (CN V) innervating the anterior part of the gland and the posterior branch to the submandibular ganglion innervating the gland posteriorly [14]. Tosios et al. described the presence of nerve cell bodies and small ganglia in the interlobular connective tissue stroma within 13 submandibular glands-generally only associated within the submandibular ganglion or the hilum of the submandibular gland [17].

Clinical significance

The parasympathetic root of the submandibular ganglion conveys the majority of salivary secretions during resting conditions. Saliva lubricates the oral cavity and is essential for mastication, digestion, and swallowing. Other components of saliva include buffers such as bicarbonate that buffer acids from dietary intake and microbial metabolism, mucins that protect the underlying epithelium, and antimicrobial proteins such as defensins and IgA that protect the body from pathogens [4-7,10]. Disorders/damage that affect the parasympathetic root result in hypersalivation or hyposalivation from the submandibular and sublingual glands. Sialorrhea or drooling is characterized by excessive secretion of saliva above the rim of the mouth. Disorders of hypersalivation are more commonly associated with impaired neuromuscular control as seen in cerebral palsy, Parkinson’s disease, and cerebrovascular accident, with dysfunction of muscles of swallowing, at times, implicated [10,26-29]. Other conditions associated with sialorrhea include infections, Wilson disease, Angelman syndrome, and poisoning [30]. Also, normal salivation could be increased by the secretory phase of the menstrual cycle, and by drugs mainly antipsychotics such as clozapine [30,31]. For instance, Sanagustin et al. [32] found that the prevalence of clozapine-induced sialorrhea was 92.3% in a study performed on 130 patients with schizophrenia spectrum disorders treated with clozapine. Moreover, other second-generation antipsychotics such as risperidone, aripiprazole, olanzapine, quetiapine, and paliperidone are less frequently related to sialorrhea [31,33]. Non-surgical management of sialorrhea includes postural correction, behavioral modification, swallowing therapy, anti-reflux and/or anti-cholinergic drugs, and botulinum toxin (BOTOX) injection within Wharton’s duct [27]. Surgical interventions have been utilized to treat severe sialorrhea; chorda tympani neurectomy as a surgical approach to treat sialorrhea results in parasympathetic denervation of the submandibular ganglion. After 4 ½ years, following transection of the chorda tympani, Chilla et al. [26] noted a decreased flow rate of submandibular gland saliva but not complete stoppage of saliva in their patients. Intraoral ablation of postganglionic parasympathetic fibers of the submandibular ganglion has been used to treat sialorrhea [27,29]. This approach may be more advantageous over transoral chorda tympani neurectomy as it preserves taste and sensory fibers, does not reduce protein contents of saliva stimulated by sympathetic innervation, and reduces damage to Wharton’s duct [27,29]. The submandibular ganglion and its roots can be damaged during removal of the submandibular gland, for instance, Preuss et al. [34] found in a study with 258 patients with sialolithiasis (46%), sialadenitis (34%), and tumors (20%), who underwent submandibular excision that the most common complications were transient palsies of the mandibular branch of the facial nerve (9%) and lingual nerve (2%).

Hyposalivation may be caused by a number of conditions that have broad etiologies including neurotransmitter receptor dysfunction, salivary gland parenchymal destruction, fluid and electrolyte imbalances, irradiation treatment for head and neck cancers, and systemic inflammatory diseases including Sjogren’s syndrome, diabetes mellitus, and amyloidosis [35]. Hyposalivation can result in xerostomia (dry mouth), halitosis, and tooth demineralization as a result of an imbalance of oral microbiota and reduced protective substances in saliva. Parasympathetic root stimulation can be affected by muscarinic receptor agonists and antagonists used in the treatment of chronic diseases such as chronic obstructive pulmonary disease (COPD), schizophrenia, and Alzheimer’s disease that result in reduced secretion of saliva from acinar cells [10,35]. Peripheral parasympathetic ganglia can be involved in Parkinson’s disease [36]. For example, Takeda et al. [36] documented intra- and extracytoplasmic Lewy bodies present in the submandibular ganglion of an 83-year-old man diagnosed with Parkinson’s disease. 

Recently, Kawashima et al. [37] found that nearly all of the postganglionic neurons in the human submandibular ganglion showed ChAT-immunoreactivity. In their study, only 18.2% of the neurons in the submandibular ganglion were positive for VIP. Earlier, Teshima et al. [38] identified a subset of neurons in the submandibular gland that stained positively for tyrosine hydroxylase.

Acknowledgements

The authors sincerely thank those who donated their bodies to science so that anatomical research could be performed. Results from such research can potentially increase mankind’s overall knowledge which can then improve patient care. Therefore, these donors and their families deserve our highest gratitude [39]. The cadaver donor used for our figures is derived from our university’s donor program.

## Conclusions

The parasympathetic root of the submandibular ganglion supplies two of the three major salivary glands. Neuromuscular disorders, systemic diseases, and drugs can affect the parasympathetic root and acinar cells in the glands it innervates, leading to hypersalivation or hyposalivation and a decrease in the quality of life of affected individuals.
